# Clinical characteristics of endometriosis with and without dysmenorrhea diagnosed by laparoscopy

**DOI:** 10.3389/fmed.2025.1635960

**Published:** 2025-09-11

**Authors:** Yi-Fen Li, Wen-Wei Li, Xiao-Hui Yang, Yue-Bo Yang, Qing-Jian Ye

**Affiliations:** Department of Gynecology, The Third Affiliated Hospital of Sun Yat-sen University, Guangzhou, Guangdong, China

**Keywords:** laparoscopy, dysmenorrhea, endometriosis, rASRM, CA125

## Abstract

**Objective:**

This study aimed to delineate the clinical characteristics of endometriosis patients with dysmenorrhea and analyze the correlations between the presence, severity, and duration of dysmenorrhea and the severity of endometriosis.

**Methods:**

In this retrospective study, a total of 489 patients with endometriosis were enrolled who had undergone laparoscopic surgery or had been diagnosed with endometriosis during surgery for benign ovarian tumors. The patients were categorized into dysmenorrhea-positive and dysmenorrhea-negative groups based on the presence of symptoms at diagnosis. Subgroup analyses were further performed within the dysmenorrhea group based on pain severity (mild/moderate/severe) and duration (years). The impact of dysmenorrhea on endometriosis severity was evaluated using clinical, surgical, and histopathological parameters.

**Results:**

The proportion of patients without dysmenorrhea was 29.4%, while the proportion of patients with dysmenorrhea was 70.6%. Patients with dysmenorrhea were found to be younger, had significantly higher CA125 levels, and had a higher surgical stage. Patients with dysmenorrhea were observed to be more likely to experience infertility and deep infiltrating nodules. Elevated CA125 levels and infiltrating nodules were found to be independent risk factors for dysmenorrhea. CA125 levels and surgical staging significantly increased with the severity of dysmenorrhea. CA199 levels increased with dysmenorrhea duration but decreased in patients with >10 years of symptoms. The longer the duration of dysmenorrhea, the more frequently adenomyosis was observed.

**Conclusion:**

The presence, severity, and prolonged duration of dysmenorrhea are strongly associated with advanced endometriosis, infertility, and adenomyosis. Young women with dysmenorrhea warrant heightened clinical suspicion for endometriosis to avoid delayed diagnosis.

## Introduction

Endometriosis (EM) is a heterogeneous disease defined by the presence of endometrial-like tissue outside the uterine cavity. It comprises three well-recognized and often interrelated subtypes: superficial endometriosis (SUP), ovarian endometrioma (OMA), and deep infiltrating endometriosis (DIE) (1). Dysmenorrhea is one of the cardinal symptoms of EM, but its association with disease progression remains controversial. Previous studies have shown that 70% of EM patients experience dysmenorrhea, while approximately 30% remain asymptomatic (2). This clinical heterogeneity suggests that dysmenorrhea may reflect distinct pathophysiological mechanisms.

In recent years, only a few studies have specifically investigated the correlation between dysmenorrhea patterns and EM. Although it is widely accepted that endometriosis is causally linked to dysmenorrhea and other symptoms such as infertility, findings regarding the association between dysmenorrhea patterns and EM severity remain inconsistent. Some studies report no correlation between dysmenorrhea and the morphological location of endometriosis, other disease-related characteristics, or disease stage and classification (3–5). Conversely, other studies suggest that patients with dysmenorrhea tend to be younger (6), present with more advanced surgical staging (7–9), and exhibit associations with DIE (9–12) and adenomyosis (13). Furthermore, the relationship between dysmenorrhea duration and cancer antigen 125 (CA125) levels or the extent of pelvic adhesions remains unclear.

This retrospective study aimed to systematically evaluate the association between dysmenorrhea characteristics (presence/absence, severity, and duration) and EM clinical parameters (the revised American Society for Reproductive Medicine (rASRM) staging, CA125 levels, comorbidities, etc.) to provide evidence for stratified clinical management.

## Materials and methods

### Study population

A total of 489 patients with pathologically confirmed endometriosis were enrolled between January 2022 and December 2023. The inclusion criteria comprised (1) age ≥18 years, (2) regular menstrual cycles, and (3) laparoscopic surgery performed either for suspected endometriosis or an incidental endometriosis diagnosis during surgery for benign ovarian tumors. The exclusion criteria comprised patients with malignancies, endocrine conditions, prior chemotherapy/radiotherapy, pregnancy, or autoimmune diseases.

### Group stratification

Patients were divided into a dysmenorrhea-negative (control) group and a dysmenorrhea-positive (case) group. Differences in serological markers, such as Anti-Müllerian Hormone (AMH), CA125, cancer antigen 199 (CA199), Human Epididymis Protein 4 (HE4), imaging findings (e.g., presence or absence of adenomyosis), and intraoperative findings were analyzed between the groups.

### Preoperative and surgical protocols

The preoperative assessment included standardized clinical and ultrasonographic evaluations. Laparoscopic procedures were performed under general anesthesia with pneumoperitoneum maintained at 13 mmHg. Surgical staging followed the rASRM criteria (14). Retrospective data collection encompassed demographics, cyst characteristics, and comorbidities.

### Subgroup analyses

The dysmenorrhea-positive cohort was further stratified by the following:

Pain severity: Graded via a visual analog scale (VAS) (15)—a 10-cm line anchored by “no pain” (0) and “worst imaginable pain” (10). Scores were categorized as follows:
Mild (VAS 1–3): Tolerable pain without daily activity impairment.
Moderate (VAS4–6): Activity-limiting pain requiring pharmacological intervention.
Severe (VAS 7–10): Debilitating pain necessitating aggressive therapy.
Pain duration: ≤1 year, 1–5 years, 5–10 years, and >10 years.

### Statistical analysis

Analyses were conducted using SPSS 21.0. Group comparisons used chi-squared tests, *t*-tests, ANOVA, Spearman’s correlation, and logistic regression (statistical significance: *p* < 0.05, two-tailed).

### Ethical compliance

The study protocol was reviewed and approved by the Institutional Review Board of the Third Affiliated Hospital of Sun Yat-sen University (Approval No. 2025-179-01). Written informed consent was obtained from all participants.

## Results

### Cohort characteristics

The study population included 345 patients (70.6%) in the dysmenorrhea group and 144 patients (29.4%) in the non-dysmenorrhea group. Among those with dysmenorrhea, pain severity distribution was as follows: mild (*n* = 204, 59.1%), moderate (*n* = 89, 25.8%), and severe (*n* = 52, 15.1%). Detailed dysmenorrhea duration data were available for 140 patients: ≤1 year (*n* = 26), 1–5 years (*n* = 41), 5–10 years (*n* = 35), and >10 years (*n* = 38). Complete clinical characteristics are presented in Table 1.

**Table 1:** A comparison of the clinical characteristics between groups with and without dysmenorrhea.

Variable	Total/category	Dysmenorrhea (*n* = 345)	Non-dysmenorrhea (*n* = 144)	*p*-value
Age (years)	489	29.95 ± 5.39	31.58 ± 6.09	0.006
BMI	489	20.3 ± 3.03	20.3 ± 2.58	0.99
AMH (ng/mL)		2.96 ± 2.12	3.13 ± 2.59	0.598
CA125 (U/mL)		128.6 ± 102.4	45.2 ± 38.7	0.001
CA199 (U/mL)		84.94 ± 144.91	75.47 ± 325.51	0.679
Sexual activity				
	No	20.0%	16.1%	0.314
	Yes	80.0%	83.9%	
Adenomyosis				
	No	87.2%	93.1%	0.081
	Yes	12.8%	6.9%	
Infertility	No	91.6%	98.4%	0.007
	Yes	8.4%	1.6%	
Laterality of ovarian cysts	Unilateral	77%	79.6%	0.56
	bilateral	23%	20.4%	
Deep infiltrating nodules				
	No	40.5%	50.7%	0.047
	Yes	59.5%	49.3%	
Douglas pouch status				
	Normal	34.8%	42.3%	
	Partial obliteration	16.7%	19.7%	0.111
	Complete obliteration	48.5%	38.0%	
rASRM score		78.5 ± 25.1	42.3 ± 18.9	0.001
rASRM stage				
	Stage I	0.3%	1.4%	
	Stage II	1.7%	3.5%	0.032
	Stage III	45.2%	50.7%	
	Stage IV	52.8%	44.4%	

### Intergroup comparisons between dysmenorrhea and non-dysmenorrhea groups

Significant differences were observed between the groups:

Age: The dysmenorrhea group (29.95 ± 5.39 years) was younger than the non-dysmenorrhea group (31.58 ± 6.09 years; *p* = 0.006).
Disease severity markers: higher CA125 levels (*p* = 0.001), elevated surgical scores (*p* = 0.001), more advanced surgical staging, a greater prevalence of deep infiltrating nodules, and higher infertility rates. Detailed data are shown in Table 2.

**Table 2:** Intergroup comparisons between the dysmenorrhea and non-dysmenorrhea groups.

Variable	Dysmenorrhea group (Mean ± SD/proportion)	Non-dysmenorrhea group (Mean ± SD/proportion)	*p*-value
CA125 (U/mL)	128.6 ± 102.4	45.2 ± 38.7	0.001
rASRM scores	78.5 ± 25.1	42.3 ± 18.9	0.001
Age (years)	29.95 ± 5.39	31.58 ± 6.09	0.006
rASRM stage IV	52.8%	44.4%	0.032
Deep infiltrating nodules	59.5%	49.3%	0.04
Infertility	8.4%	1.6%	0.007

There were no significant differences between the two groups in terms of body mass index (BMI); the presence or absence of sexual activity; the number of pregnancies and deliveries; serum levels of CA199, HE4, and AMH; unilateral vs. bilateral ovarian cysts; the presence or absence of posterior fornix obliteration; or the presence or absence of adenomyosis.

The presence or absence of dysmenorrhea showed no significant association with lesion locations (e.g., non-endometriotic ovarian cysts, pelvic sidewall, intestinal serosal surface, and ureteral serosal surface) at any site. Detailed data are shown in Table 3.

**Table 3:** Dysmenorrhea status in different lesion locations.

Lesion location	Ovary	Pelvic wall, intestinal surface	Ureteral surface	*p*-value
Non-dysmenorrhea group (proportion)	50.0%	45.8%	4.2%	0.076
Dysmenorrhea group (proportion)	42.0%	55.9%	2.1%	

### Intergroup comparisons by dysmenorrhea severity (mild, moderate, severe)

The average age of the mild dysmenorrhea group was 29.87 ± 5.43 years, the average age of the moderate dysmenorrhea group was 29.88 ± 5.43 years, and the average age of the severe dysmenorrhea group was 30.38 ± 5.24 years. There was no significant age difference among the three groups of patients. There was a statistically significant difference (*p* = 0.001) in CA125 levels and surgical scores among the three groups (see Table 4).

**Table 4:** Intergroup comparisons by dysmenorrhea severity.

Variable	Mild (Mean ± SD/proportion)	Moderate (Mean ± SD/proportion)	Severe (Mean ± SD/Proportion)	*p*-value
CA125 (U/mL)	58.2 ± 40.1	135.7 ± 88.3	228.2 ± 120.5	0.001
rASRM scores	60.3 ± 20.5	85.2 ± 22.7	110.4 ± 30.8	0.001

No significant differences were observed among the three groups in terms of body mass index (BMI); the presence or absence of sexual activity; number of pregnancies and deliveries; serum levels of CA199, HE4, and AMH; unilateral vs. bilateral ovarian cysts; the presence or absence of deep infiltrating nodules (DINs); the presence or absence of posterior fornix obliteration; or the presence or absence of adenomyosis. The severity of dysmenorrhea was weakly associated with lesion location (*p* = 0.251).

### Intergroup differences in menstrual pain duration

Based on the duration of dysmenorrhea, patients were stratified into the following four groups: ≤1 year, 1–5 years, 5–10 years, and >10 years. The duration of dysmenorrhea was found to be positively correlated with CA125 and surgical staging (*p* < 0.001). The level of CA199 increased with the duration of dysmenorrhea but decreased in patients with more than 10 years of dysmenorrhea (≤1 year group: 29.54 ± 29.92 U/mL, 1–5 years group: 38.85 ± 174.89 U/mL, 5–10 years group: 141.91 ± 194.28 U/mL, >10 years group: 59.8 ± 73.32 U/mL, *p* = 0.029). Patients with longer dysmenorrhea had a higher incidence of uterine adenomyosis (*p* = 0.046).

No significant differences were observed among the four groups in terms of body mass index (BMI); the presence or absence of sexual activity; the number of pregnancies and deliveries; serum levels of CA199, HE4, and AMH; unilateral and bilateral ovarian cysts; deep infiltrating nodules; or cul-de-sac obliteration. Due to the small number of cases with lesions located on the ureteral surface, we reclassified lesion locations as ovarian lesions or lesions at other sites. No significant difference was observed in the duration of dysmenorrhea between the two groups (*p* = 0.501).

### Multifactor analysis

In the multivariable logistic regression analysis with dysmenorrhea (presence/absence) as the dependent variable, CA125 elevation (adjusted OR = 4.32, 95% CI 2.35–7.23; *p* < 0.001) and DIE nodules (adjusted OR = 2.89, 95% CI 1.45–5.78; *p* < 0.003) showed significant associations. See Table 5 for complete regression results.

**Table 5:** Multivariate logistic regression analysis of dysmenorrhea-associated risk factors.

Variable	OR value	95%CI	*p*-value
CA125 (>35 U/mL)	4.12	[2.35, 7.23]	<0.001
Deep infiltrating nodules	2.89	[1.45, 5.78]	0.003
CA199	0.999	[0.99, 1.00]	0.337
HE4	0.997	[0.96, 1.03]	0.882
Laterality of ovarian cysts	1.536	[0.54, 4.34]	0.418
Adenomyosis	1,415	[0.41, 4.80]	0.578
Douglas pouch status	1.048	[0.55, 1.19]	0.887
Sexual activity	0.957	[0.37, 2.51]	0.928

## Discussion

Dysmenorrhea, defined as intense visceral pain during menstruation, represents a form of pain and is characterized as “an unpleasant sensory and emotional experience associated with actual or potential tissue damage, or described in terms of such damage.” Its perception may involve both the mind and body, severely impacting patients’ mental health (16). Dysmenorrhea is classified as primary dysmenorrhea or secondary dysmenorrhea (17). Primary dysmenorrhea is defined as dysmenorrhea occurring in the absence of pelvic pathology; secondary dysmenorrhea arises from pelvic pathology or recognized medical conditions, with endometriosis being the primary cause of secondary dysmenorrhea in adolescents (18, 19).

The majority of endometriosis patients develop dysmenorrhea during adolescence or young adulthood, predominantly at ≤25 years of age (18). This symptom is often highly underestimated, perceived as a normal and transient phenomenon in young individuals (20). Moreover, despite severe pain symptoms, a diagnosis in young endometriosis patients is frequently missed during examinations such as ultrasounds, leading to delayed diagnosis. This diagnostic oversight may result from multiple factors, primarily because endometriotic lesions are typically small and not clearly visible on ultrasound or laparoscopy (21). This study pioneered the simultaneous analysis of three dysmenorrhea dimensions (presence, severity, and duration). It revealed that the dysmenorrhea group exhibited a younger age of onset (29.95 ± 5.39 vs. 31.58 ± 6.09 years, *p* = 0.006), higher CA125 levels (128.6 ± 102.4 vs. 45.2 ± 38.7 U/mL, *p* = 0.001), and higher rASRM scores (78.5 ± 25.1 vs. 42.3 ± 18.9, *p* = 0.001), along with a higher incidence of deep infiltrating nodules (DINs) and infertility, aligning with findings from previous studies (6–12). Patients with higher dysmenorrhea severity showed elevated CA125 levels and advanced surgical staging, while longer dysmenorrhea duration correlated with a higher coexistence of adenomyosis.

Previous studies indicate that individuals experiencing frequent dysmenorrhea have an 18.874-fold higher risk of adult-onset endometriosis compared to those experiencing occasional dysmenorrhea. Those developing dysmenorrhea >12 months after menarche had a 5.257-fold higher risk of endometriosis than those with an onset at 6 months post-menarche. Adolescent dysmenorrhea predicts a higher future endometriosis risk (22, 23). A significant correlation exists between ovarian endometriosis and adenomyosis. The prevalence rates in young women are 25.0% (endometriosis) and 46.0% (adenomyosis), with a coexistence rate of 50%. Adolescent endometriosis often represents an early phenotype of adenomyosis, posing diagnostic challenges. External adenomyosis is more common in young, nulliparous women with a history of endometriosis (24).

Dysmenorrhea is a risk factor for adenomyosis, and the findings of this study strengthen the hypothesis of a progressive and common disease course in the natural history of endometriosis, which initially manifests as symptoms, then as signs of pelvic adhesions, and finally progresses to adenomyosis, ovarian endometriosis, or deep infiltrating endometriosis (25). Dysmenorrhea is also associated with infertility rates in endometriosis patients (26). Preoperative CA125 and CA199 levels combined with pain scores may predict pelvic endometriosis and could potentially be used in infertility evaluations (27). Therefore, the clinical significance of this study lies in recommending an early endometriosis risk assessment for young, nulliparous patients with dysmenorrhea, using dysmenorrhea severity, duration, and CA125 levels as markers of disease severity. An early clinical diagnosis of endometriosis without invasive surgery can promptly relieve pain, prevent central sensitization and chronic pain, reduce infertility risks, and improve quality of life (28).

Additionally, this study analyzed the relationship between different endometriosis lesion locations (e.g., ovarian endometriosis, pelvic wall, intestinal surface, or ureteral endometriosis) and the presence/absence of dysmenorrhea, severity of dysmenorrhea, and duration of dysmenorrhea. The results indicated that, although deep infiltrating endometriosis (DIE) was associated with the presence of dysmenorrhea, there was no correlation between different endometriosis lesion locations and dysmenorrhea. This finding may be attributed to the overly general recording of lesion locations during surgery; future studies should more meticulously document lesion locations and counts.

This study also found that CA199 levels increased with the duration of dysmenorrhea, suggesting that CA199 may serve as a marker for the severity of endometriosis, consistent with previous findings. High CA199 expression may correlate with poor patient prognosis (28–31). However, a paradoxical decrease in CA199 levels was observed in the group with >10 years of dysmenorrhea duration, potentially due to fluctuations in CA199 levels with the menstrual cycle and reduced secretion from advanced fibrotic lesions. The specific mechanism underlying this phenomenon remains to be further investigated.

Methodologically, this study used a multivariable logistic regression analysis to control confounders, surpassing the univariate analyses commonly used in previous research. Limitations include the following: (1) subjective, recall-based dysmenorrhea scoring, (2) unassessed histological indicators (e.g., nerve fiber density), and (3) a lack of postoperative pain follow-up data. Future studies should integrate quantitative sensory testing (QST) and dynamic CA125 monitoring. (4) Another limitation of this study was the limited number of cases complicated by adenomyosis, which is likely due to the study’s retrospective design and the reliance on preoperative imaging for adenomyosis diagnosis. Further prospective studies are warranted to validate the relationship between endometriosis and adenomyosis and their contribution to dysmenorrhea.

## Conclusion

This study confirms that the presence, severity, and duration of dysmenorrhea are significantly associated with endometriosis severity, specifically manifested by dysmenorrhea patients exhibiting more active biological characteristics (elevated CA125 levels and a higher incidence of DIE) and more advanced rASRM staging. In clinical practice, early assessment of young patients with severe dysmenorrhea may help prevent disease progression.

## Data Availability

The raw data supporting the conclusions of this article will be made available by the authors, without undue reservation.
