# Correction: Clinical characteristics of endometriosis with and without dysmenorrhea diagnosed by laparoscopy

**DOI:** 10.3389/fmed.2026.1887446

**Published:** 2026-06-23

**Authors:** Yi-Fen Li, Wen-Wei Li, Xiao-Hui Yang, Yue-Bo Yang, Qing-Jian Ye

**Affiliations:** Department of Gynecology, The Third Affiliated Hospital of Sun Yat-sen University, Guangzhou, Guangdong, China

**Keywords:** laparoscopy, dysmenorrhea, endometriosis, rASRM, CA125

There were several mistakes in Table 1 as published.

**Table 1:** A comparison of the clinical characteristics between groups with and without dysmenorrhea.

Variable	Total/category	Dysmenorrhea (*n* = 345)	Non- dysmenorrhea (*n* = 144)	*P*-value
Age (years)	489	29.95 ± 5.39	31.58 ± 6.09	0.006
BMI	489	20.3 ± 3.03	20.3 ± 2.58	0.99
AMH (ng/mL)		2.96 ± 2.12	3.13 ± 2.59	0.598
CA125 (U/mL)		128.6 ± 102.4	45.2 ± 38.7	0.001
CA199 (U/mL)		84.94 ± 144.91	75.47 ± 325.51	0.679
Sexual activity				
	No	20.0%	16.1%	0.314
	Yes	80.0%	83.9%	
Adenomyosis				
	No	87.2%	93.1%	0.081
	Yes	12.8%	6.9%	
Infertility				
	No	91.6%	98.4%	0.007
	Yes	8.4%	1.6%	
Laterality of ovarian cysts				
	Unilateral	77%	79.6%	0.56
	bilateral	23%	20.4%	
Deep infiltrative nodules				
	No	40.5%	50.7%	0.047
	Yes	59.5%	49.3%	
Douglas pouch status				
	Normal	34.8%	42.3%	0.111
	Partial obliteration	16.7%	19.7%	
	Complete obliteration	48.5%	38.0%	
rASRM score		78.5 ± 25.1	42.3 ± 18.9	0.001
rASRM stage	Stage I	0.3%	1.4%	0.032
	Stage II	1.7%	3.5%	
	Stage III	45.2%	50.7%	
	StageIV	52.8%	44.4%	

In the original Table 1, the data for “Deep infiltrative nodules” were incorrectly reported. Specifically, in the Non-dysmenorrhea group, the proportion for “No” was listed as 80.0% and for “Yes” as 20.0%. The correct values should be 50.7% for “No” and 49.3% for “Yes.” Additionally, the *P*-value was originally shown as 0.04, but the correct *P*-value is 0.047.

Furthermore, in the row for “rASRM stage,” the original table showed “Stage I” in the Total/Category column, but it should simply read “Stage” as the category header. Also, the percentage for Stage II in the Non-dysmenorrhea group was originally 2.8%, which should be corrected to 3.5%. For Stage IV in the Dysmenorrhea group, the original value was 58.2%, which should be corrected to 52.8%.

The corrected Table 1 appears below.

There was a mistake in Table 3 as published.

In the original Table 3, under the “dysmenorrhea Group (Proportion)” row, the percentage for “Ureteral Surface” was listed as 2.0%. The correct value should be 2.1%. The corrected Table 3 appears below.

**Table 3:** Dysmenorrhea status according to lesion location.

Lesion location	Ovary	Pelvic wall, intestinal surface	Ureteral surface	*P*-value
Non-dysmenorrhea group (Proportion)	50.0%	45.8%	4.2%	0.076
Dysmenorrhea group (Proportion)	42.0%	55.9%	2.1%	

The original version of this article has been updated.

